# Forced MyD88 signaling in microglia impacts the production and survival of regenerated retinal neurons

**DOI:** 10.3389/fcell.2024.1495586

**Published:** 2024-11-20

**Authors:** Jordan E. Rumford, Ailis Grieshaber, Samantha Lewiston, Jordan L. Reed, Samuel S. Long, Diana M. Mitchell

**Affiliations:** ^1^ Department of Biological Sciences, University of Idaho, Moscow, ID, United States; ^2^ Department of Computer Science, University of Idaho, Moscow, ID, United States; ^3^ Formerly North Idaho College, Coeur d’Alene, ID, United States; ^4^ Business and Computer Science Division, Lewis-Clark State College, Lewiston, ID, United States

**Keywords:** microglia, retina, regeneration, inflammation, MyD88, NFkB, Müller glia, zebrafish

## Abstract

Inflammation and microglia appear to be key factors influencing the outcome of retinal regeneration following acute retinal damage. Despite such findings, direct connection of microglia-specific inflammatory factors as drivers of regenerative responses in the retina are still not defined, and intracellular pathways activated to stimulate such signals from microglia are currently unknown. We became interested in MyD88 regulation in microglia because transcriptomic datasets suggest *myd88* could be regulated temporally in zebrafish microglia responding to damage in the central nervous system. MyD88 is an intracellular molecular adaptor that initiates signaling cascades downstream of several innate immune receptors, and probably most well-known for inducing gene expression of pro-inflammatory factors. Using zebrafish, which spontaneously regenerate retinal neurons after acute retinal damage, we studied the effects of overactivation of MyD88 signaling in microglia and macrophages on the Müller glia-mediated regenerative response. Our results indicate that increased MyD88 signaling in microglia/macrophages impacts the initial response of Müller glia entering a regenerative response after acute, neurotoxin-induced retinal damage to inner retinal neurons. In addition, increased MyD88 signaling in microglia/macrophages resulted in reduced survival of inner retinal neurons in regenerated retinas. This work supports the idea that temporal control of inflammatory signaling is a key component in the production of MG-derived progenitors yet further indicates that such control is important for differentiation and survival of regenerated neurons.

## Introduction

Retinal regeneration upon acute damage occurs spontaneously in zebrafish, but not in mammals. Due to their intrinsic regenerative capacity, zebrafish serve as an important model organism to study retinal regeneration with translatable findings (Todd et al., 2021; Jorstad et al., 2020; Jorstad et al., 2017; Ueki et al., 2015; Pollak et al., 2013; Lahne et al., 2020; Rueda et al., 2019; Yao et al., 2016; Wohlschlegel et al., 2023). It is now recognized that Müller glial responses underlie the disparate regenerative outcomes in zebrafish compared to mammals. In zebrafish retina, Müller glia (MG) respond to acute retinal damage by re-entering the cell cycle to produce a proliferating pool of neuronal progenitors (Bernardos et al., 2007; Nagashima et al., 2013; Fausett and Goldman, 2006; Fimbel et al., 2007; Thummel et al., 2008). However, in mammals Müller glia instead enter a gliotic response with severely limited ability to produce neuronal progenitors (Bringmann et al., 2009). Inflammation has emerged as a key regulator of the zebrafish Müller glial response to retinal damage (Zhang et al., 2020; White et al., 2017; Silva et al., 2020; Nagashima and Hitchcock, 2021; Iribarne and Hyde, 2022; Bludau et al., 2024). Cellular sources of inflammatory signals presumably include the microglia, which are the resident immune cells of the vertebrate retina. In support of such a role, microglia express many inflammatory cytokines (Mitchell et al., 2019; Hoang et al., 2020) and microglial manipulations have been shown to have effects on the Müller glial regenerative response in mouse (Todd et al., 2020), chick (Fischer et al., 2014), and zebrafish (White et al., 2017; Conedera et al., 2019). In addition, immunosuppressant treatments alter the kinetics of zebrafish retinal regeneration (White et al., 2017; Silva et al., 2020; Bludau et al., 2024). Despite these findings, direct connection of microglia-specific inflammatory factors as drivers of MG responses is still not well established. Though certain cytokines may be involved (Zhao et al., 2014; Lu and Hyde, 2024; Nelson et al., 2013), the cell types producing these cytokines and the timing of expression remain poorly defined. Moreover, intracellular pathways activated to stimulate such signals from microglia are currently unknown.

Temporal control of inflammatory signals and the downstream responses that are induced are likely important for transitioning from quiescence to the initial damage response then to a regenerative phase. Indeed, the regulation of Nfkb transcriptional activity, which is induced by inflammatory signaling through numerous cell surface and intracellular receptors, likely influences the outcome of Müller glial responses in the mouse and chick (Palazzo et al., 2020; Palazzo et al., 2022). Numerous innate immune receptors detect ligands such as those released from damaged cells and activate intracellular pathways culminating on NfkB signaling and cytokine production, and cytokine signaling may continue to regulate inflammatory responses downstream of the initial stimulus.

Toll-like receptors (TLRs) and IL-1 family receptors are membrane bound innate immune receptors known to activate a shared intracellular molecular adaptor, MyD88 (Deguine and Barton, 2014; Warner and Núñez, 2013). Beyond these canonical activators of MyD88, MyD88 may also participate in other immune response pathways (Chen et al., 2022; Sun and Ding, 2006; Liu et al., 2011). MyD88 activates signaling cascades resulting in transcriptional and cellular responses in cells responding to extracellular signals including damage-associated molecules (Deguine and Barton, 2014; Warner and Núñez, 2013). Nfkb is one of several transcription factor families activated downstream of MD88 signaling (Deguine and Barton, 2014). The MyD88 pathway is an important response mechanism in macrophages and plays a role in microglial responses (Esen and Kielian, 2006). Several cytokines found to be important in regulating regenerative responses (Lu and Hyde, 2024; Hasegawa et al., 2017; Tsarouchas et al., 2018; Nguyen-Chi et al., 2017) are known to be induced downstream of MyD88 signaling and Nfkb activation. Further, constitutively active MyD88 signaling has effects on cell proliferation and survival in cancer (Wang et al., 2014; Ngo et al., 2011).

In this paper, we focused on the effects of overactivation of MyD88 signaling in microglia and macrophages on the Müller glial regenerative response in the zebrafish retina. We became interested in MyD88 regulation in microglia because transcriptomic datasets suggest it could be regulated temporally in microglia responding to damage in the central nervous system (Mitchell et al., 2019; Oosterhof et al., 2017), Figure 1. We hypothesized that forcing inflammatory signals from microglia via sustained MyD88 signaling during retinal regeneration would alter the outcome of Müller glia-mediated regenerative responses. To test our hypothesis, we generated transgenic zebrafish in which forced MyD88 signaling occurs cell-selectively in microglia and macrophages. Using these transgenic fish, we examined the MG-mediated regenerative response triggered upon neurotoxin-induced death of inner retinal neurons. We analyzed the acute damage response, production and proliferation of MG-derived progenitors, and early regeneration of inner retinal neurons. Our results indicate that regulation of inflammatory signals produced by microglia and macrophages partly drive the temporal transition from inflammatory/proliferative to regenerative responses. In addition, our results indicate that the downstream signals from MyD88 activation in microglia/macrophages impact the survival of regenerated retinal neurons.

**FIGURE 1:**
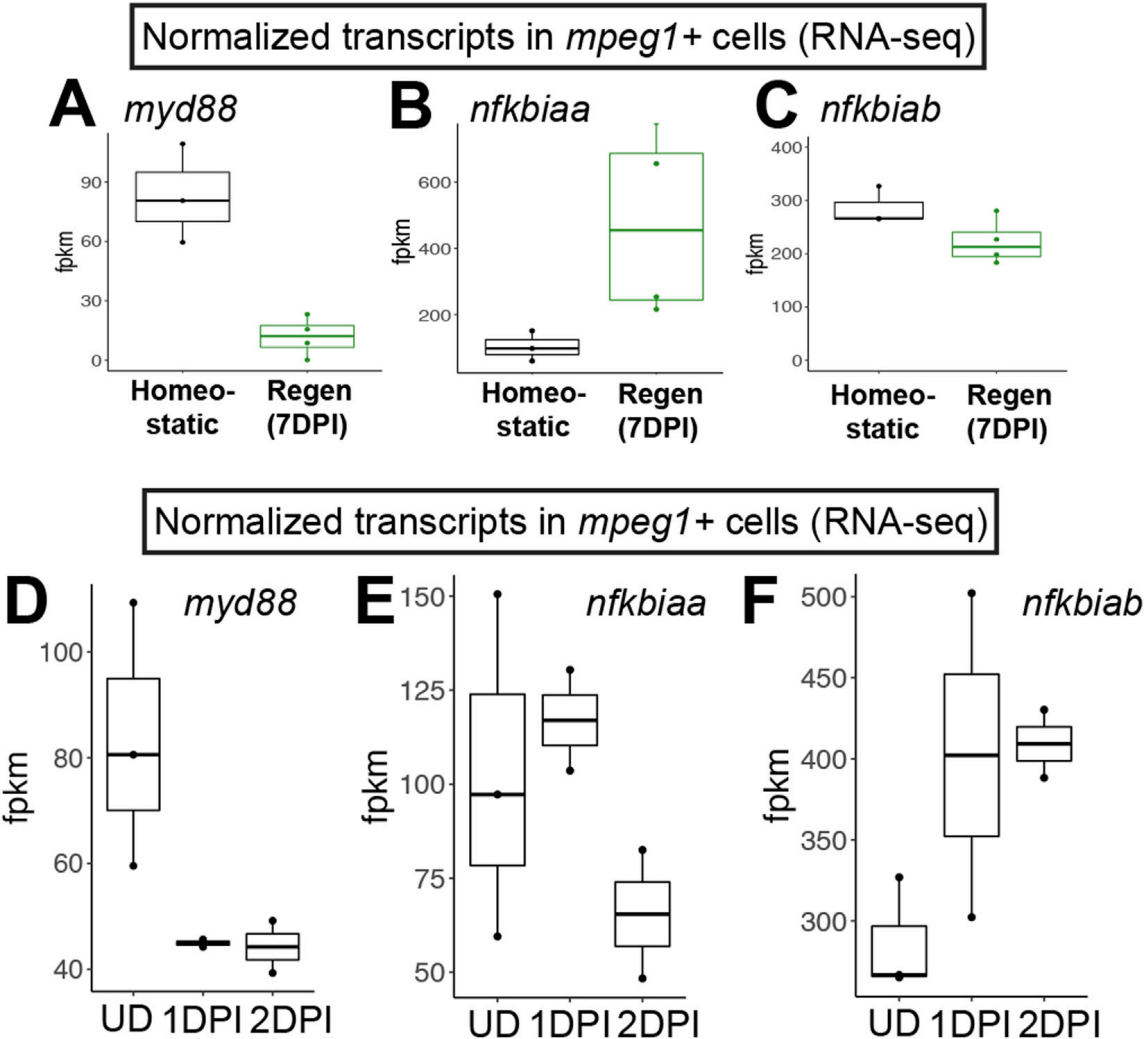
RNA-seq indicates downregulation of *myd88* in *mpeg1*+ cells during retinal regeneration and upon CNS damage. Normalized transcript counts (fragments per kilobase million, fpkm) of *myd88* **(A)**, *nfkbiaa* **(B)**, *nfkbiab* **(C)** in *mpeg1*:GFP+ cells isolated from undamaged zebrafish brain [homeostatic (Oosterhof et al., 2017)] or regenerating zebrafish retinas [Regen (7DPI) (Mitchell et al., 2019)]. Normalized transcript counts (fragments per kilobase million, fpkm) of *myd88* **(D)**, *nfkbiaa* **(E)**, *nfkbiab* **(F)** in *mpeg1*:GFP+ cells isolated from undamaged (UD) or acutely damaged zebrafish brain at 1 or 2 days post injury (1DPI, 2DPI) using transcriptome data from (Oosterhof et al., 2017). Fish used in the retina sequencing study were 10–12 months old (Mitchell et al., 2019); fish used in the brain study were 3 months old (Oosterhof et al., 2017).

## Materials and methods

### Zebrafish

Procedures using zebrafish were performed at the University of Idaho in compliance with IACUC (Institutional Animal Care and Use Committee) approved protocols. Zebrafish (*Danio* rerio) were maintained on a 14:10 light:dark cycle in 28°C recirculating, monitored system water. Zebrafish were housed and propagated according to (Westerfield, 2007). Zebrafish lines used include *mpeg1*:GFP (Ellett et al., 2011) (gl22Tg, originally obtained from Zebrafish International Resource Center, ZIRC), *mpeg1*:mCherry (Ellett et al., 2011) (gl23Tg, originally obtained from ZIRC), 6xHSA.NFKB:EGFP (Kanther et al., 2011) (nc1Tg, obtained from ZIRC, referred to throughout the manuscript as NfkB::gfp), and *mpeg1*:myd88-2A-mCherry (uoi2505Tg, generated in-house, described below). All lines were bred to (at least 8 generations) or generated using an in-house “wildtype” strain that was originally obtained from Scientific Hatcheries (now Aquatica Tropicalis). Zebrafish used were of both sexes and ranged in age from 4–21 months. Prior to tissue collection, fish were euthanized by extended immersion in tricaine solution (0.25 mg/mL).

Generation of the *mpeg1*:myd88-2A-mCherry transgenic line (uoi2505Tg): The zebrafish *myd88* cDNA sequence (NCBI NM_212814.2) was amplified from cDNA generated from mRNA isolated from whole zebrafish retinas. Primers in the PCR reaction were designed to selectively amplify *myd88* and included restriction sites for EcoR1 and NotI (Forward: 5′-TAA​GCA​GAA​TTC​ATG​GCA​TCA​AAG​TTA​AGT​ATA​GAC​CA-3′, Reverse: 5′- TAA​GCA​GCG​GCC​GCG​GGC​AGT​GAA​AGT​GCT​TTG​GC-3′). The PCR amplicon was excised from the agarose gel after electrophoresis and purified using the NEB Monarch Gel Extraction Kit, then digested with EcoR1 and NotI (NEB). The amplicon was ligated into pME-MCS (also previously digested with EcoR1 and NotI) using Promega T4 DNA Ligase. After transformation and plating, colonies were selected for liquid cultures and plasmid minipreps purified using the Qiagen QIA Prep Spin Minprep Kit. Plasmids with correct size inserts were sequenced by Sanger sequencing. The transgenesis vector was created by using Gateway LR reaction with p5E-mpeg1.1 (Don et al., 2017) (plasmid 75023 obtained from Addgene), pME-*myd88*, 543-p3E-2A-mCherrypA (Villefranc et al., 2013) (plasmid 2603 obtained from Addgene), and pDEST-Tol2CG2 as described in (Kwan et al., 2007). After transformation and colony selection, transgenesis constructs with the proper inserts and orientation were identified by Restriction Enzyme digestions. Single cell stage zebrafish embryos were injected with ∼0.5–1 nL of the transgenesis construct mixed with Tol2 mRNA at 25 ng/μL final concentration. F0 founders were selected based on expression of GFP fluorescent hearts, grown to adults, then outcrossed with wildtype fish. F1 fish were identified by germline inheritance of GFP+ hearts, reared to adults, then again outcrossed to wildtype to obtain F2 with stable integration. F2 lines were examined and selected for mCherry fluorescence in microglia and ∼50% transgene segregation upon outcross to non-transgenic partners. Fish were bred to F3 generation and later for experiments. Fish of the uoi2505Tg transgenic line were healthy and viable to adult age with similar survival rates to other lines used in this study.

### Retinal lesion

Retinal lesions were performed by intravitreal injection of the neurotoxin ouabain (ouabain octahydrate, Sigma-Aldrich) as described in previous publications (Mitchell et al., 2019; Mitchell et al., 2018) and extensively detailed in (Mitchell and Stenkamp, 2023), using 40–80 µM ouabain working solutions prepared in 0.65% sterile saline (NaCl) to induce the death of inner retinal neurons. Injections were performed with a blunt-end calibrated 10 μL Hamilton syringe, 26 s gauge, point style 3. Lesions were unilateral and only the right eye was injected. Saline injected retinas served as additional controls; these fish received an intravitreal injection of 0.65% sterile saline (NaCl, vehicle) solution in the right eye. Prior to and during the procedure, fish were continuously anaesthetized with tricaine in water solution (0.25 mg/mL). Immediately after the procedure, fish were returned to clean tanks with clean system water for revival and recovery. All fish receiving such injections fully recovered from the procedure.

### Tissue fixation and processing for retinal cryosections for TUNEL and immunofluorescent staining

Ocular enucleation was performed using fine forceps, whole eyes were then transferred to PBS (phosphate-buffered saline), followed by cornea puncture and lens removal. Eyes were fixed in phosphate buffered, 4% paraformaldehyde containing 5% sucrose at 4°C for 16–20 h with constant rocking. Eyes were then washed in a graded series of phosphate-buffered solution (pH = 7.4) of 5% sucrose to 20% sucrose. The following day, tissues were washed in a 1:2 solution of OCT embedding medium (Sakura Finetek) and phosphate buffered, 20% sucrose at room temperature (RT) for 30 min. After the wash, eyes were embedded in fresh 1:2 solution of OCT embedding medium (Sakura Finetek) and phosphate buffered, 20% sucrose and frozen by immersion in 2-methylbutane supercooled with liquid nitrogen. After freezing solid, the tissue blocks were stored at −20°C for at least 24 h prior to sectioning. Eyes were sectioned at 10 µM thickness using a Lieca CM3050 cryostat and mounted onto glass slides (FisherBrand Superfrost Plus Microscope Slides). Tissue sections underwent overnight desiccation and were then stored at −20°C until use.

### Tissue fixation and processing of whole retinas for immunofluorescent staining

Fish were dark adapted for approximately 12 h and ocular enucleation was performed using fine forceps. Whole eyes were then transferred to PBS, followed by cornea puncture and lens removal. Retinas were isolated from the whole eye cup and retinal pigmented epithelium (RPE) was removed prior to PBS rinses. Retinas were fixed in phosphate buffered, 4% paraformaldehyde in PBS at 4°C for 16–24 h with constant rocking. Post fixation, retinas were washed several times in PBS with 0.01% Triton-X-100 (PBST) and dehydrated in a graded series of methanol washes prior to storing at −20°C in 100% methanol until use.

### Immunofluorescence staining of retinal cryosections and whole retinas

Retinal cryosections were thawed in a humidified chamber for 10 min at RT followed by a 1 h blocking period with 1% normal donkey serum (NDS), 0.1% sodium azide in PBS-Triton-X-100 (PBST). Retinal cryosections were stained overnight at 4°C with primary antibodies in an antibody dilution buffer, PBST, 1% NDS, and 0.1% sodium azide. Following incubation, retinal cryosections were washed in PBST at RT for 30 min then stained with secondary antibodies and 4′,6-diamidino-2-phenylindole (DAPI) diluted in antibody dilution buffer at RT for at least 1 h. Following secondary incubation, retinal cryosections underwent a second 30-min PBST wash and multiple PBS rinses. Coverslips were mounted using an antifade mounting media (Vectashield Vibrance).

Whole retinas were rehydrated in a series of PBST washes at RT prior to a 1 h blocking period with 1% normal donkey serum (NDS) and 0.1% sodium azide in PBST. Retinas were stained over night at 4°C with primary antibodies in an antibody dilution buffer (PBST, 1% NDS, and 0.1% sodium azide). Following incubation, retinas underwent a series of PBST washes at RT prior to the secondary staining. Secondary antibodies and DAPI were diluted in antibody dilution buffer, staining was performed at RT for 2 h. Retinas were then washed in a series of PBST, followed by a PBS wash. Four radial incisions were made in the retina to allow for a flat mount. Coverslips were mounted using an antifade mounting media (Vectashield Vibrance).

Primary antibodies and dilutions used are as follows. Rabbit polyclonal anti-zebrafish L-plastin (Guyader et al., 2008) (1:10,000, a kind gift from Dr. Michael Redd), rat anti-PCNA (1:200, Chromotek, Cat 16d10, Lot 090428013AB), mouse 4C4 antibody (1:100, used as hybridoma supernatant, hybridoma 7.4.C4 sourced from Sigma-Aldrich used to stain zebrafish microglia, Cat 92092321, Lot 15B027), mouse anti-GFAP (1:100, ZRF1 obtained from ZIRC, ZDB-ATB-081002-46), mouse anti-Glutamine Synthetase (1:100, BD Biosciences, Cat 610517, Lots 1340268, 3166939), mouse anti-Neurolin/DM-GRASP/Alcama (1:500, Zn-5 (also known as Zn-8), obtained from ZIRC, ZDB-ATB-081002-22), chicken anti-GFP (1:1000, Abcam, Cat AB13970, Lot 1018753-23), Rabbit anti-HuC/D (1:200, AbCam Cat AB210554, Lots GR3445684-5, GR3208493-3). Detection of PCNA required a citrate antigen retrieval prior to immunolabeling. Antigen retrieval was performed as detailed in (Lovel and Mitchell, 2023). Post antigen retrieval, retinal cryosections entered a blocking step using a 20% NDS, 0.1% sodium azide, in PBS-Triton-X-100 for 1 h at RT.

Secondary antibodies and dilutions used are as follows. Donkey anti-mouse FITC, donkey anti-rabbit FITC, donkey anti-mouse Cy3, donkey anti-rabbit Cy3, donkey anti-mouse Alexa-Fluor647, donkey anti-rabbit Alexa-Fluor647, donkey anti-Chicken Alexa-Fluor488, donkey anti-Chicken Alexa-Fluor647 (Jackson ImmunoResearch), donkey anti-rat Alexa-Flour647 (Thermo Scientific), and goat anti-mouse Cy7 (AAT Bioquest) were used at 1:200 dilution. DAPI was used at 1:1000 dilution in all stains and added to the secondary antibody solution.

TUNEL (Terminal deoxynucleotidyl transferase dUTP nick end labeling) staining (Roche, Fluorescein Green Kit and TMR Red Kit, Cats 11684795910, 12156792910) of retinal cryosections was performed using Roche manufacturer’s instructions. Prior to immunolabeling, retinal cryosections were thawed in a humidified chamber at RT for 10 min and washed in PBS for 20 min. Retinal cryosections underwent permeabilization for 2 min in a chilled 0.1% Triton-X-100, 0.1% sodium citrate solution on ice. Slides were then washed twice in PBS for 5 min prior to undergoing the TUNEL reaction. TUNEL reactions were performed at 37°C for 3 h in a humidified chamber following the manufacturer’s instructions. Following the reaction, slides were rinsed in PBS for 5 min three times. Immunolabeling procedure was performed following TUNEL labeling, beginning at the blocking step.

### Microscopy and image acquisition

Images were captured using a Nikon Andor X1 spinning disk confocal microscope or Nikon CrestOptics X-Light confocal microscope, both equipped with BSI Express 16-bit sCMOS camera and utilizing Nikon Elements software. Imaging was performed using a Plan Apo λ 20X Air 0.75 NA DIC or CFI APO LWD 40x water immersion 1.15 NA λ S DIC N2 objectives. Z stacks were acquired in 2–3 µm intervals. For cryosections, one control retinal cryosection was used to determine optimal image acquisition settings for laser power and exposure. For whole retinas, a small segment of one control retina was used for selection of optimal image conditions. Image viewing and processing was performed with Nikon Elements software and ImageJ (Fiji).

### Quantification of cell types in retinal cryosections from confocal images

Quantification of cells across timepoints was performed using ImageJ (Fiji). Leukocytes (including microglia), TUNEL+ cells, PCNA+ cells, and HuC/D+ inner retinal neurons were quantified within the retina in individual z stacks in the image. Cells that were L-plastin+ were counted as leukocytes and counts were reported based on the region of interest (ROI) area or to a specified curvilinear distance of the ROI. Total number of TUNEL+ cells were counted and normalized to the ROI area or curvilinear distance. For certain timepoint(s) L-plastin+ cells were also scored for nuclear PCNA signal. HuC/D+ inner retinal neurons were counted as those with HuC/D+ signal surrounding DAPI+ nucleus. Counts were performed using images from central retinal regions and excluded regions at the peripheral edges and optic nerve head (ONH).

Quantification of total PCNA+ DAPI+ cells was achieved using a partially automated Python script run through a Windows command shell. The script is available upon request. Image data was read using Python package Nd2reader. ROI selection required non-automated, user defined input, using a data visualization library called Matplotlib (Hunter, 2007). Image filters (unsharp, Gaussian blur, and edge detection) were applied to the images using the Sci-Kit Image library (Walt et al., 2014). Multiple thresholding techniques (multi-Otsu, Otsu, and local) using The Open-CV library were then applied to perform further segmentation of cells. Signal from channels associated with DAPI or PCNA less than 15 square pixels in area were excluded to avoid quantification of artifacts. To obtain cell counts, signals from both DAPI and PCNA channels were overlayed using a binary logical “AND” algorithm, implemented using Numpy (Harris et al., 2020). Objects with PCNA and DAPI co-localization meeting the criteria were reported based on curvilinear distance in the retinal cryosection.

### RNA isolation, cDNA synthesis, and quantitative PCR (RT-qPCR)

RNA was extracted from whole retinas; either undamaged or following injections of either saline or ouabain. Retinas dissected were processed individually, there was no sample pooling. Retinas were immediately transferred to and homogenized in the RNA lysis solution using a handheld homogenizer (Bio-Gen Series PRO200, Pro Scientific), then stored at −80°C for a maximum of 1 week, until RNA extraction. RNA extraction was performed using the NucleoSpin^®^ RNA kit (Macherey-Nagel), following the manufacturer’s protocol. RNA was quantified and quality checked using a NanoDrop™ One Microvolume UV-Vis Spectrometer (Thermo Scientific). Following RNA extractions, cDNA was synthesized using SuperScript IV First-Strand cDNA Synthesis Reaction kit (Invitrogen) using random hexamers. cDNA samples were stored at −20°C until qPCR reaction set up.

Gene-specific primers for qPCR and their sources are shown in Table 1. Primers were verified for specificity by NCBI Primer Blast vs. GRCz11. Amplification of qPCR reactions were performed using PowerTrack SYBR-Green Master Mix (Applied Biosystems), 1–2.5 ng cDNA template per reaction and run with a QuantStudio™ 3 Real-Time PCR System (Thermo Fisher). The chosen reference gene was *18s*, which returned consistent Ct readings (inter-sample ranges within ∼1–1.4^−ΔΔCT^ of each other) between all samples. Other reference genes (*bact2*, *elf1a*, *gapdh*) were deemed unsuitable due to variability in Ct readings >2^−ΔΔCT^ between samples. RT-qPCR reactions underwent 40 amplification cycles. No-template control wells were included for each primer set and melt curves were checked for clean and specific peaks after each run. Fold change was calculated using the delta delta Ct method.

**TABLE 1:** Primer sequences used in qPCR.

Primer	Sequence 5′ → 3′	Primer sequence source
*18 s* F’ *18 s* R’	GAACGCCACTTGTCCCTCTA GTTGGTGGAGCGATTTGTCT	Sherpa et al. (2014)
*ascl1a* F’ *ascl1a* R’	ATCTCCCAAAACTACTCTAATGACATGAACTCTAT CAAGCGAGTGCTGATATTTTTAAGTTTCCTTTTAC	Mitra et al. (2022)
*atoh7* F’ *atoh7* R’	TGCAAGAGAACGAAAGAGAGTGC TTCCGAAGCCGGTCGAA	Sherpa et al. (2014)
*brn3b* F’ *brn3b* R’	AAAGTCGCACCGGGAGAAA TTCTCGGCCCCGTTGA	Sherpa et al. (2014)
*fgf8a* F’ *fgf8a* R’	GCTCCAAAACCAGGCAACA CTTGGGCAACCTCTTCATGAA	Sherpa et al. (2014)
*gfap* F’ *gfap* R’	CTAAGCCAGACTTGACCGCT TTACGATTGGCTGCATCCGT	Mitchell et al. (2019), Thiel et al. (2022)
*GFP* F’ *GFP* R’	AAGGGCATCGACTTCAAGGA TGATGCCGTTCTTCTGCTTG	Seda et al. (2019)
*irg1* F’ *irg1* R’	CACTGCCAGCACATGTATGCT CACCTTGGCGCCCATAGA	Hall et al. (2013)
*lin28a* F’ *lin28a* R’	TAACGTGCGGATGGGCTTCGGATTTCTGTC ATTGGGTCCTCCACAGTTGAAGCATCGATC	Ramachandran et al. (2010)
*mCherry* F’ *mCherry* R’	GTGATGAACTTCGAGGACGGCG CTTCTTCTGCATTACGGGGCCG	This study
*pcna* F’ *pcna* R’	ATGATCTCGTGTGCCAAGGATGG CTGCAATTTTGTACTCAACCACTAG	Mitra et al. (2022)
*il1b F*’ *il1b R*’	GCTCATGGCGAACGTCATCC CGCACTTTCAAGTCGCTGCT	Lu and Hyde (2024)
*tnfa F*’ *tnfa R*’	TCACGCTCCATAAGACCCAG GATGTGCAAAGACACCTGGC	Tsarouchas et al. (2018)
*tnfb F*’ *tnfb R*’	CCTCAGACCACGGAAAAGT GCCCTGTTGGAATGCCTGAT	Silva et al. (2020)
*il6 F*’ *il6 R*’	ACACTCAGAGACGAGCAGTTTG ACCACGTCAGGACGCTGTAG	Lu and Hyde (2024)
*il10 F*’ *il10 R*’	GCACTCCACAACCCCAATCG TGGCAAGAAAAGTACCTCTTGCAT	Lu and Hyde (2024)
*mmp9 F*’ *mmp9 R*’	TGATGTGCTTGGACCACGTAA ACAGGAGCACCTTGCCTTTTC	Silva et al. (2020)
*mmp13a F*’ *mmp13a R*’	ATGGTGCAAGGCTATCCCAAGAGT GCCTGTTGTTGGAGCCAAACTCAA	Hillegass et al. (2007)
*mmp14 b F*’ *mmp14 b R*’	AATGGCAAGGCGTTCCAGACAACA CTCTCACGTTCCCGGTCTCGGTCA	Zlatanova et al. (2023)

### Statistical analysis

Statistics were performed in the R coding environment. For pairwise comparisons, non-parametric Welch’s tests were performed. For multiple groups, Kruskal–Wallis test was used, followed by Conover’s posthoc. Statistically significant differences are annotated in the figures.

## Results

### Increased inflammatory signaling by forcing expression of *myd88* in microglia upon retinal damage

We previously interrogated gene expression of *mpeg1*+ cells (microglia/macrophages) isolated from regenerating zebrafish retinas at 7 days post injury (DPI) following ouabain-induced cytotoxic lesion using RNA-sequencing (Mitchell et al., 2019). Examining normalized gene expression of *mpeg1*+ cells in our dataset (Mitchell et al., 2019) compared to that of *mpeg1*+ cells isolated from undamaged zebrafish brain in a separate published study (Oosterhof et al., 2017) indicated that microglia in regenerating retinas downregulate expression of *myd88* (Figure 1A). We also found that *mpeg1*+ cells in regenerating retinas upregulate *nfkbiaa* and maintain high levels of *nfkbiab*, which encode two inhibitors of Nfkb activation (Figures 1B,C), suggesting that there is inhibition of the MyD88/Nfkb signaling pathway in microglia during retinal regeneration. In addition, by examining *myd88* mRNA levels for other samples in the Oosterhof et al. (2017) transcriptomic study, we found that *mpeg1*+ cells downregulate *myd88* by 1 day post brain injury (Figure 1D). This downregulation of *myd88* is accompanied by upregulation of *nfkbiab* through 2 days post injury, with less obvious changes in *nfkbiaa* (Figures 1E,F). Collectively, these results suggest that there is temporal regulation of the MyD88 signaling pathway in microglia/macrophages after central nervous system damage.

**FIGURE 2:**
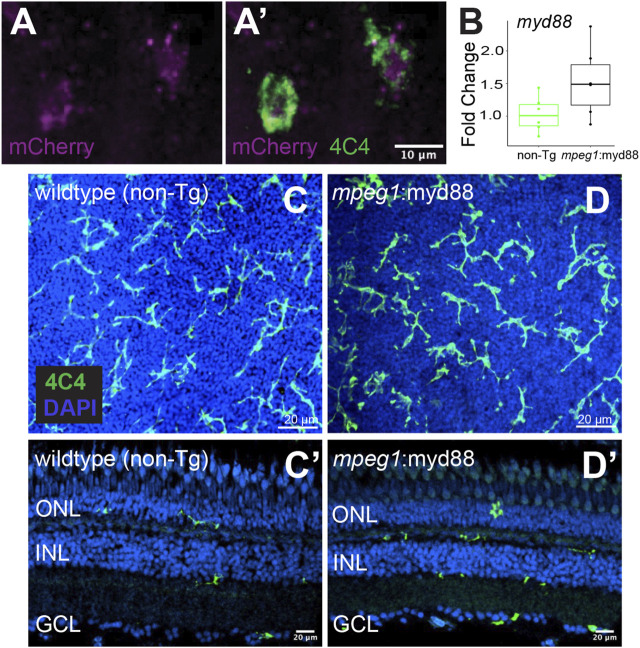
Creation of transgenic zebrafish with forced *myd88* expression in microglia/macrophages. Tol2 transgenesis followed by several generations of outcrossing was used to generate a zebrafish line in which the *mpeg1* promoter drives expression of *myd88* cDNA followed by a 2A-mCherry sequence. **(A, A’)** mCherry fluorescence in 4C4+ microglia visualized from *mpeg1*:myd88-2A-mCherry zebrafish eyes/retinas at 3 days post fertilization (dpf). Through the remainder of the manuscript, we refer to this line as *mpeg1*:myd88 for simplicity. **(B)** Fold change of *myd88* transcripts measured by RT-qPCR in whole retinas from non-transgenic (non-Tg) and *mpeg1*:myd88 adult fish. Each dot represents result of one single adult retina. Differences were not statistically significant (*p* = 0.08). **(C, D)** Microglia stained and visualized with the 4C4 antibody using whole, flat mounted adult zebrafish retinas. Nuclei stained with DAPI. **(C’, D’)** Retinal cryosections (adult) stained and visualized with 4C4 antibody and DAPI. ONL = outer nuclear layer, INL = inner nuclear layer, GCL = ganglion cell layer.

Given that MyD88/Nfkb signaling is known to induce gene expression of inflammatory factors, and the recent evidence that inflammation regulates retinal regeneration in zebrafish (Zhang et al., 2020; White et al., 2017; Silva et al., 2020; Iribarne and Hyde, 2022; Bludau et al., 2024), we hypothesized forcing inflammatory signals from microglia via sustained MyD88 signaling during retinal regeneration would alter the outcome of Müller glia-mediated regenerative responses. As an experimental tool to test our hypothesis, we generated a transgenic zebrafish line in which the *myd88* cDNA sequence is expressed under control of the *mpeg1* promoter, linked with viral T2A peptide-mCherry (*mpeg1*:*myd88*-2A-mCherry). The stable line selected for experiments displayed mCherry fluorescence in microglia in larvae (Figures 2A,A’) though fluorescent mCherry expression in adult retinas was extremely weak/difficult to detect. In adult retinas, mRNA levels of *myd88* in whole adult retinas were slightly elevated in some samples compared to non-transgenics (but not statistically significant, Figure 2B). Microglia are a fraction of all retinal cells, and reporter protein expression from viral 2A polycistronic expression systems can vary (Ryan and Drew, 1994; Kim et al., 2011). Collectively, these data suggest low expression of the transgene by microglia in the undamaged adult retina. In these *mpeg1*:*myd88*-2A-mCherry (here after referred to as *mpeg1*:myd88) transgenic fish, microglia differentiated and populated the adult retina (Figures 2C,D’) and retinal lamination was found to be grossly normal (Figures 2C’, D’).

**FIGURE 3:**
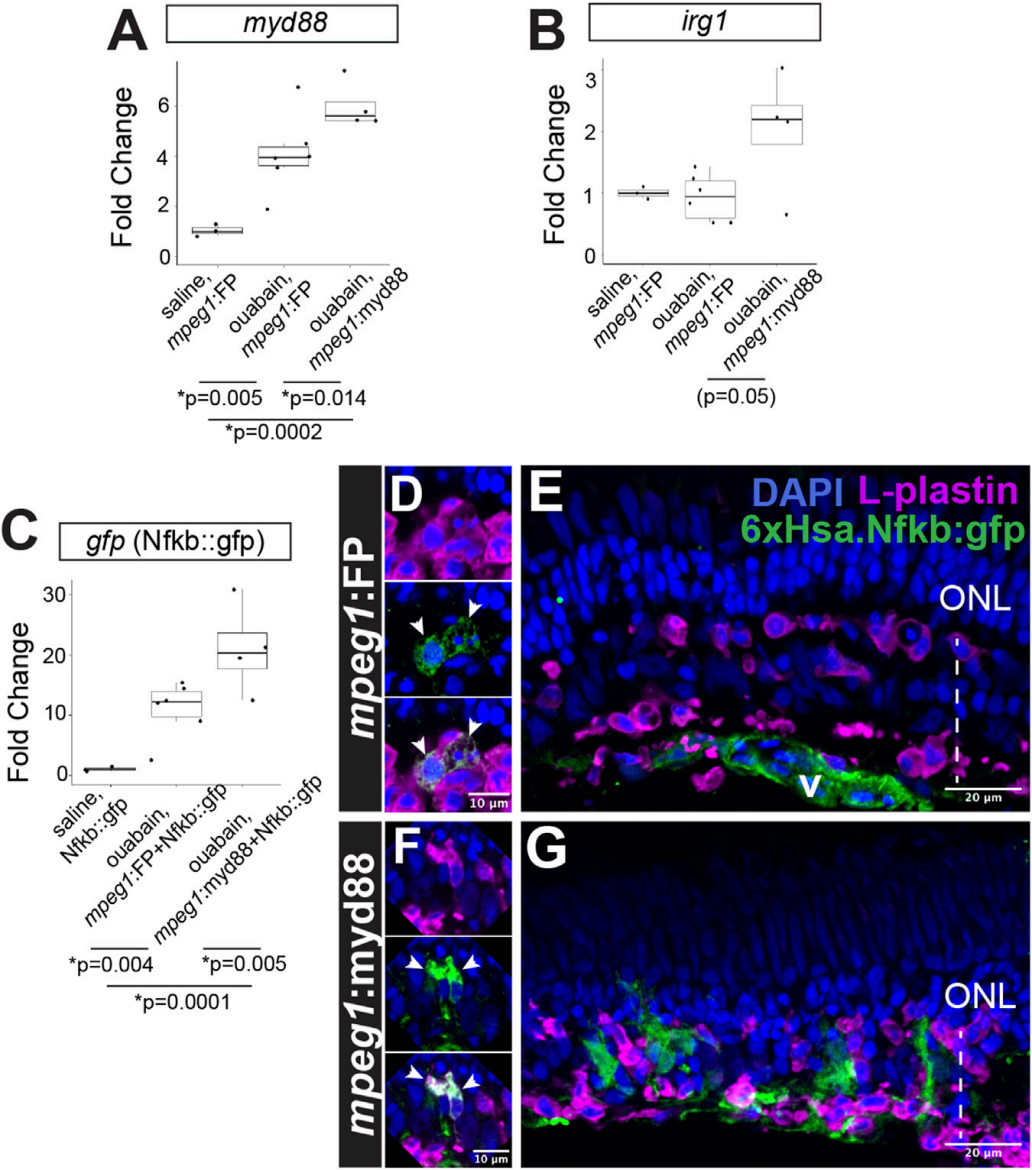
Prolonged inflammatory signaling in retinas with forced *myd88* signaling in microglia/macrophages. RT-qPCR was used to measure transcripts for **(A)** *myd88*, **(B)** *irg1*, and **(C)** *gfp* after intravitreal injection of ouabain. **(A, B)** Fold change of each transcript in ouabain injected samples relative to saline injected samples. **(C)** Fold change of *gfp* in retinas also carrying the Nfkb::gfp transcriptional reporter. **(A–C)** *p*-values shown below the graphs indicate statistically significant differences between the indicated groups (Kruskal–Wallis, followed by Conover’s posthoc). **(D, E)** Visualization of GFP in Nfkb::gfp reporter line at 4 days post-ouabain injection (4DPI). **(D)** Enlarged panels show GFP signal detected in a subset of responding L-plastin+ cells within the damaged inner retina (arrowheads). **(E)** GFP expression also detective in vascular (v) structures. **(F, G)** Visualization of GFP in *mpeg1*:myd88 transgenics also carrying Nfkb::gfp reporter. **(F)** Enlarged panels show strong GFP expression visible in a subset of responding L-plastin+ cells within the damaged inner retina (arrowheads). **(G)** GFP expression also seen in regions of the inner nuclear layer that is consistent with Müller glia and Müller glia-derived progenitors at 4DPI. ONL = outer nuclear layer; the vertical dotted line indicates inner retinal region damaged by ouabain.

To determine the effects of *myd88* overexpression in microglia/macrophages on retinal regeneration, we utilized a system of retinal damage by intravitreal injection with the neurotoxin ouabain (Mitchell et al., 2019; Mitchell et al., 2018; Mitchell and Stenkamp, 2023). As controls for *mpeg1*:myd88 fish, we used *mpeg1*:GFP or *mpeg1:*mCherry transgenics, to control for expression of *mpeg1*-driven transgenes. Throughout the manuscript we refer to samples from these *mpeg1*-reporter lines as “*mpeg1*:FP” since their results represent outcomes following retinal damage to which to compare samples with *mpeg1*-driven *myd88* expression. At 4 days post ouabain injection (4DPI), *myd88* in whole, homogenized retinal samples was increased in *mpeg1*:FP samples compared to saline injected samples (Figure 3A), suggesting that regulation of *myd88* expression is dynamic and could involve other cell types besides microglia/macrophages. Consistent with this, published single cell RNA-seq datasets show *myd88* expression is not limited to microglia and is detectable in cell types such as Müller glia and retinal pigment epithelium in undamaged and degenerating retinas (Santhanam et al., 2023). As expected, *mpeg1*:myd88 retinas had increased expression of *myd88* relative to *mpeg1*:FP, consistent with forced expression via the integrated *mpeg1*-driven transgene (Figure 3A). Further, we were able to reliably detect expression of mCherry mRNA at 4DPI in samples from *mpeg1*:mCherry and *mpeg1*:myd88-2A-mCherry by RT-qPCR (Supplementary Figure S1), confirming transgene expression and suggesting that retinal damage may trigger higher levels of transgene expression in the *mpeg1*:myd88 line.

Prolonged/forced expression of *myd88* in *mpeg1*+ cells was also associated with increased expression of *irg1* (also called *acod1*), which is associated with inflammatory macrophage/microglia activation state (Li et al., 2023; Thomas et al., 2006) and interacts with MyD88 (Chen et al., 2022), Figure 3B. Consistent with increased (or prolonged) expression of *myd88* and induction of the downstream signaling pathway, we also detected increased activity of Nfkb transcriptional activity in the 6xHsa.Nfkb::gfp reporter line by qPCR (Figure 3C). Examining 6xHsa.Nfkb::gfp (referred to as Nfkb::gfp) retinal cryosections for GFP reporter fluorescence revealed that a subset of L-plastin+ leukocytes, which represent microglia/macrophages as determined by our previous studies (Mitchell et al., 2019; Mitchell et al., 2018), express the Nfkb transcriptional reporter at 4DPI in both *mpeg1*:FP (*mpeg1*:mCherry used in these experiments) and *mpeg1*:myd88 tissue (Figures 3D,F), with apparently stronger signal in the *mpeg1*:myd88 line (Figures 3D,F). Throughout the *mpeg1*:FP retinal sections, besides the subset of microglia/macrophages, GFP was detected in regions/structures putatively representing the vasculature but not significantly found in other retinal cells or regions (Figure 3E). However, in *mpeg1*:myd88 retinal sections, patches of GFP signal were visible in the regenerating inner nuclear layer (INL) that did not colocalize with L-plastin and are consistent with Müller glia and/or newly generated, MG-derived progenitors (Figure 3G).

We also measured expression of selected cytokines (*il1b, il6, tnfa, tnfb, il10*) by RT-qPCR (Supplementary Figure S2) in samples prepared from whole retina RNA at 4DPI after saline or ouabain injection. These cytokine genes were selected because they have been shown to be regulated in zebrafish retinas after injury and during regeneration (Silva et al., 2020; Lu and Hyde, 2024; Nelson et al., 2013), and some may have a role in regenerative responses (Lu and Hyde, 2024). Of the cytokines evaluated, expression of *il1b* and *tnfa* were increased in both *mpeg1*:FP and *mpeg1*:myd88 at 4DPI ouabain compared to saline at similar levels. Although increased relative to saline controls in both lines, *tnfb* had lower expression in *mpeg1*:myd88 samples compared to *mpeg1*:FP. The cytokine *il10* was upregulated only in *mpeg1*:myd88 retinas compared to both *mpeg1*:FP and saline controls, possibly reflecting counter-balance to pro-inflammatory signals. We also examined expression of selected matrix metalloproteinase genes (Supplementary Figure S2) because inflammatory signals can modulate expression and activity of this class of enzymes. We examined *mmp9* because it has been shown to have a role in retinal regeneration in zebrafish (Silva et al., 2020). We also examined expression of *mmp13a* and *mmp14b* because these were shown to be expressed in a subpopulation of microglia in published single-cell transcriptome datasets from zebrafish retina after injury (Hoang et al., 2020). Of these, *mmp13a* trended towards higher expression in *mpeg1*:myd88 samples compared to *mpeg1*:FP and saline controls. While *mmp9* and *mmp14b* both were increased in ouabain damaged samples from both lines compared to saline controls, their expression levels were not different between *mpeg1*:FP and *mpeg1*:myd88 samples. Collectively, these RT-qPCR results indicate that forced/prolonged expression of *myd88* in microglia/macrophages alters microglia/macrophage activation state and influences inflammatory signaling within the regenerating retina.

### Effects of forced MyD88 expression in microglia/macrophages on response to retinal damage

**FIGURE 4:**
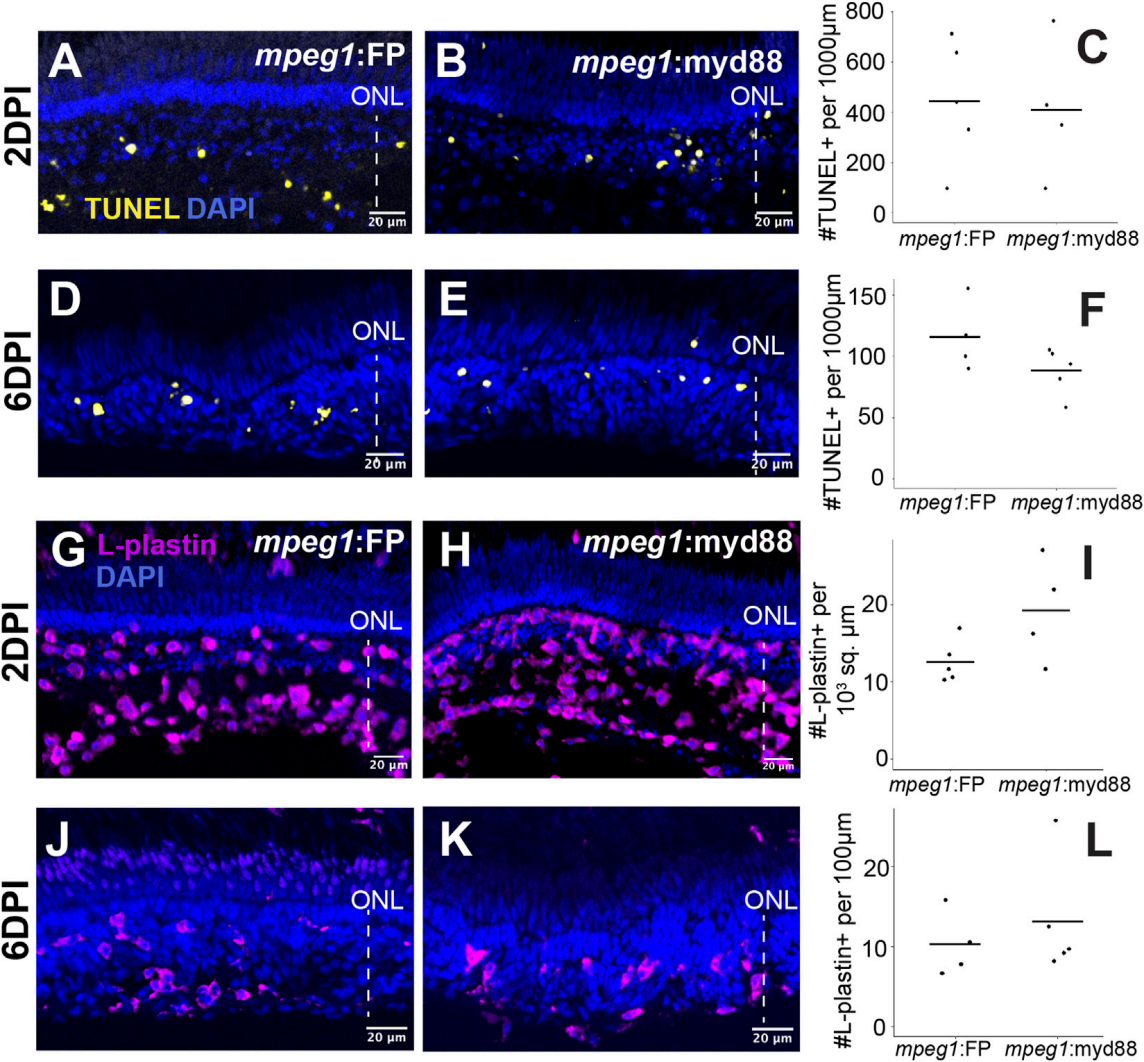
Leukocyte responses and cell death levels in acutely damaged and regenerating retinas. **(A, B)** TUNEL staining of retinal cryosections from *mpeg1*:FP or *mpeg1*:myd88 fish at 2 days post ouabain injection (2DPI, acute damage). **(C)** TUNEL counts in retinal cryosections from *mpeg1*:FP or *mpeg1*:myd88 fish at 2DPI. **(D, E)** TUNEL staining of retinal cryosections from *mpeg1*:FP or *mpeg1*:myd88 fish at 6DPI. **(F)** TUNEL counts in retinal cryosections from *mpeg1*:FP or *mpeg1*:myd88 fish at 6DPI. No statistically significant differences were found between *mpeg1*:FP and *mpeg1*:myd88 groups (Welch’s test). **(G, H)** Retinal cryosections at 2DPI stained and imaged for L-plastin and DAPI. **(I)** Quantification of L-plastin+ cells at 2DPI. **(J, K)** Retinal cryosections at 6DPI stained and imaged for L-plastin and DAPI. **(L)** Quantification of L-plastin+ cells at 6DPI. No statistically significant differences were found between *mpeg1*:FP and *mpeg1*:myd88 groups (Welch’s test). ONL = outer nuclear layer; the vertical dotted line indicates inner retinal region damaged by ouabain.

We examined lesioned retinas from control and *mpeg1*:myd88 fish for microglia/macrophage responses and the cell death marker TUNEL, at 2 and 6DPI (Figure 4), to determine if cell death and/or responding microglia/macrophage were affected by the overexpression of *myd88*. These timepoints were selected because they represent induced inner retinal neuron death and microglial/macrophage responses (2DPI) and when Müller glia/neuronal progenitors are actively proliferating (6DPI). The number of TUNEL+ cells were no different at 2 and 6DPI (Figures 4A–F), indicating that cell death was not increased (or decreased) as a result of prolonged *myd88* expression. Further, apparent regions of retinal damage remained similar between *mpeg1*:FP and *mpeg1*:myd88 retinas with regions of degeneration predominantly localized to the inner retina (Figures 4A,B,D,E).

To label microglia/macrophages, we used the pan-leukocyte marker L-plastin. We selected this marker because our previous work showed that essentially all leukocytes responding to ouabain lesion at these timepoints are microglia and macrophages (Mitchell et al., 2019; Mitchell et al., 2018), and staining retinal cryosections with the commonly used antibody “4C4” revealed that only a subset of L-plastin+ cells at 2DPI stain with 4C4 (Supplementary Figure S3). Interestingly, the antigen recognized by the 4C4 antibody is thought to be LGals3bp (Rovira et al., 2022), suggesting that this gene is differentially expressed in microglia/macrophages responding to widespread retinal lesion. Alternatively, this marker may differentially label populations of microglia *versus* infiltrating macrophages. We therefore selected L-plastin to label responding microglia/macrophages for the remainder of this study. At 2DPI, L-plastin+ microglia/macrophages were found to densely populate the damaged inner retina in both *mpeg1*:FP and *mpeg1*:myd88 samples (Figures 4G,H), consistent with our previous work (Mitchell et al., 2019; Mitchell et al., 2018). Numbers of L-plastin+ cells were slightly elevated in *mpeg1*:myd88 retinal sections compared to *mpeg1*:FP, but this difference was not statistically significant (Figure 4I). L-plastin+ cells were detected in the regenerating inner retina in both lines at 6DPI (Figures 4J,K) and on average at similar numbers (Figure 4L). These results indicate that there was not a significant increase in the initial responding microglia/macrophage numbers and forced expression of *myd88* does not result in increased neuronal death or degeneration.

**FIGURE 5:**
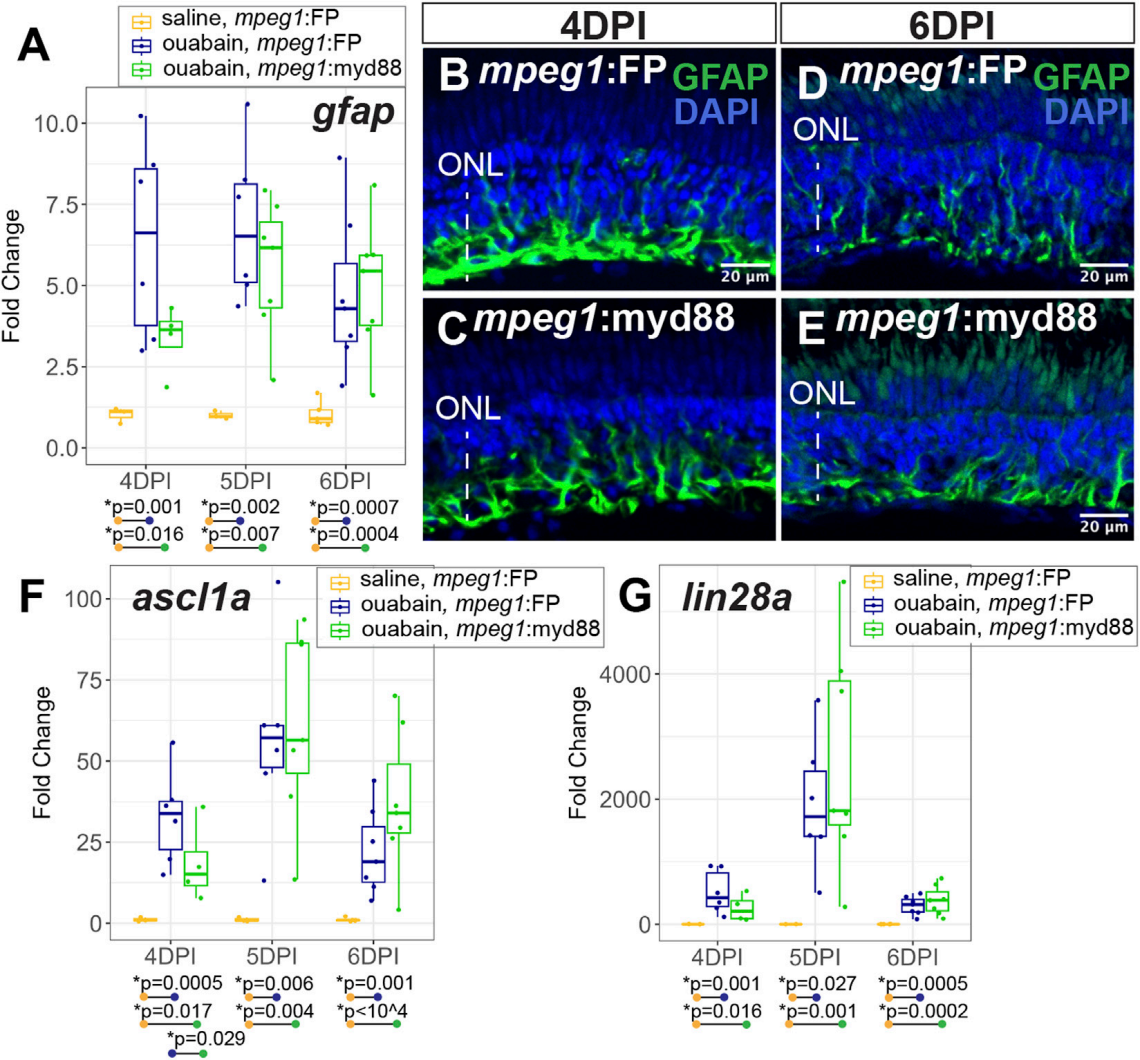
Analysis of Müller glia reactivity and induction of stem-like genes in damaged retinas. **(A)** RT-qPCR was used to examine upregulation of *gfap* in *mpeg1*:FP and *mpeg1*:myd88 retinas at 4-, 5-, or 6-days post ouabain injection (4-6DPI), fold change was determined relative to saline injected samples. Statistically significant differences between groups are shown by the *p*-values reported at the bottom of the plot. B-E. Retinal cryosections stained for GFAP and DAPI at 4DPI **(B, C)** or 6DPI **(D, E)**. ONL = outer nuclear layer; the vertical dotted line indicates inner retinal region damaged by ouabain. **(F, G)** RT-qPCR was used to examine upregulation of *ascl1a* **(F)** and *lin28a* **(G)** in *mpeg1*:FP and *mpeg1*:myd88 retinas; fold change was determined relative to saline injected samples. Statistically significant differences between groups are shown by the *p*-values reported at the bottom of the plot (Kruskal–Wallis, followed by Conover’s posthoc).

During a regenerative response in the zebrafish retina, Müller glia transiently enter a gliotic phase after retinal damage (Thomas et al., 2016). We therefore measured expression of *gfap*, an intermediate filament protein known to be upregulated in gliotic neuroglia (Bringmann et al., 2006). At 4-6DPI ouabain injection, *gfap* was increased in both *mpeg1*:FP and *mpeg1*:myd88 retinas compared to saline injected retinas (Figure 5A). At 4DPI, levels of *gfap* in damaged *mpeg1*:FP retinas were higher than those in *mpeg1*:myd88 retinas; although this difference was surprisingly not statistically significant the trend was strong (Figure 5A). At 5-6DPI, levels of *gfap* were similar between damaged *mpeg1*:FP and *mpeg1*:myd88 samples (Figure 5A). Immunostaining for GFAP in retinal cryosections was largely consistent with the RT-qPCR results (Figures 5B–E). Signal intensity appeared stronger in *mpeg1*:FP compared to *mpeg1*:myd88 retinas at 4 DPI (Figures 5B,C), but was similar at 6DPI (Figures 5D,E).

Induction of the gene *ascl1a* and its transcriptional target *lin28a* are known to be induced in zebrafish retina during a regenerative response (Ramachandran et al., 2010; Fausett et al., 2008; Ramachandran et al., 2011). Expression of these genes are thought to induce a stem-like state of Müller glia and the MG-derived progenitors. Both *ascl1a* and *lin28a* were induced in *mpeg1*:FP and *mpeg1*:myd88 retinas from 4–6 days post ouabain damage (Figures 5F,G), with a transient peak in expression at ∼5DPI. However, levels of *ascl1a* were lower in *mpeg1*:myd88 retinas compared to *mpeg1*:FP at 4DPI (Figure 5F). By 6DPI, levels of *ascl1a* were slightly elevated in *mpeg1*:myd88 samples compared to *mpeg1*:FP (trending but not statistically significant at 6DPI, Figure 5F). Trends of expression from 4 to 6DPI were similar for *lin28a* in terms of induction of expression in both *mpeg1*:FP and *mpeg1*:myd88 lines. Though a trend but not statistically significant, *lin28a* transcripts were lower in *mpeg1*:myd88 compared to *mpeg1*:FP at 4DPI (Figure 5G). It is also worth noting that *lin28a* was only just detectable in saline injected samples, with high Ct readings and with 1-2 saline samples returning “non-detect” in one or more technical replicates. This would be expected given that this gene is not strongly expressed in undamaged retinas. When also considering results described for *gfap* above, these stem-like gene expression differences suggest a delay in reactivity and induction of a stem-like state in MG/MG-derived progenitors in *mpeg1*:myd88 retinas compared to *mpeg1*:FP.

**FIGURE 6:**
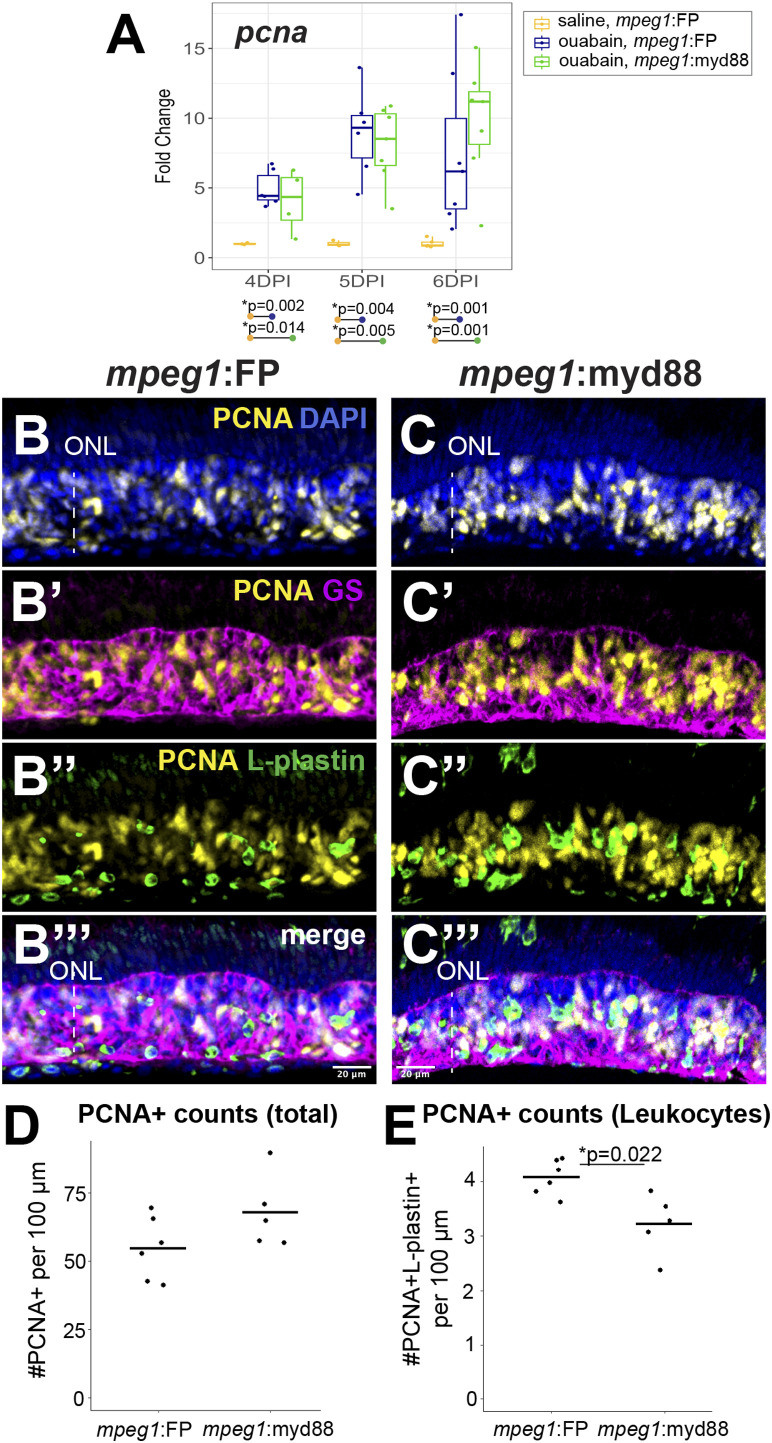
Analysis of proliferative response in damaged retinas. **(A)** RT-qPCR was used to examine upregulation of *pcna* transcript in *mpeg1*:FP and *mpeg1*:myd88 retinas at 4-, 5-, or 6-days post ouabain injection (4-6DPI); fold change was determined relative to saline injected samples. Statistically significant differences between groups are shown by the *p*-values reported at the bottom of the plot (Kruskal–Wallis, followed by Conover’s posthoc). **(B, C’’’)** Retinal cryosections were stained for PCNA, Glutamine Synthetase (GS), L-plastin, and DAPI. Selected overlays for *mpeg1*:FP and *mpeg1*:myd88 samples are shown in **(B-B’’’)** and **(C-C’’’)**. ONL = outer nuclear layer; the vertical dotted line indicates inner retinal region damaged by ouabain. **(D)** Quantification of total PCNA+ cells in retinal cryosections; PCNA+ DAPI+ nuclei were counted for this analysis. **(E)** Quantification of PCNA+ L-plastin+ cells in retinal cryosections; L-plastin+ cells with PCNA+ DAPI+ nuclei were counted for this analysis. Statistically significant difference is indicated by the shown *p*-value (Welch’s test).

We examined proliferation of MG/MG-derived progenitors by examining induction of the S-phase marker PCNA (Figure 6). We again analyzed retinas at 4, 5, and 6DPI since these timepoints are when MG/MG-derived progenitors are actively dividing and readily labeled by PCNA expression (Nagashima et al., 2013; Fimbel et al., 2007; Mitchell et al., 2019; Mitchell et al., 2018). As expected, RT-qPCR revealed that both *mpeg1*:FP and *mpeg1*:myd88 retinas strongly increased *pcna* expression relative to saline controls from 4-6DPI (Figure 6A). Levels of *pcna* were similar between *mpeg1*:FP and *mpeg1*:myd88 samples at 4 and 5DPI. At 6DPI, levels of *pcna* in *mpeg1*:myd88 retinas trended higher than *mpeg1*:FP (though this was not statistically significant), Figure 6A. Immunostaining for PCNA at 6DPI showed dense clusters of PCNA+ cells in the regenerating inner retina in both *mpeg1*:FP and *mpeg1*:myd88 retinas (Figures 6B,C). Quantifications of total PCNA+ cells in the inner retina revealed a trend consistent with that of RT-qPCR results at 6DPI, where numbers of PCNA+ cells were increased in the *mpeg1*:myd88 retinas compared to *mpeg1*:FP (Figure 6D, though again not statistically significant). Some of the PCNA+ cells were surrounded by Glutamine Synthetase (GS) signal, which is a known marker of Müller glia, and therefore likely representing S-phase+ MG (Figures 6B’,B’’). However, many of the PCNA+ cells in the inner retina at 6DPI are likely MG-derived progenitors because most PCNA+ nuclei did not co-localize with GS (Figure 6B’, B’’) or L-plastin (Figures 6C’,C’’). To better understand differences in total PCNA quantifications, we counted PCNA+ L-plastin+ cells (likely representing dividing microglia/macrophages). In contrast to total PCNA counts, the numbers of PCNA+ L-plastin+ cells were reduced in *mpeg1*:myd88 retinas compared to *mpeg1*:FP (Figures 6C’, C’’). These results further suggest that there are more PCNA+ MG/MGPCs in *mpeg1*:myd88 retinas at 6DPI compared to *mpeg1*:FP.

### Effects of forced MyD88 expression in microglia/macrophages on regeneration of inner retinal neurons

**FIGURE 7:**
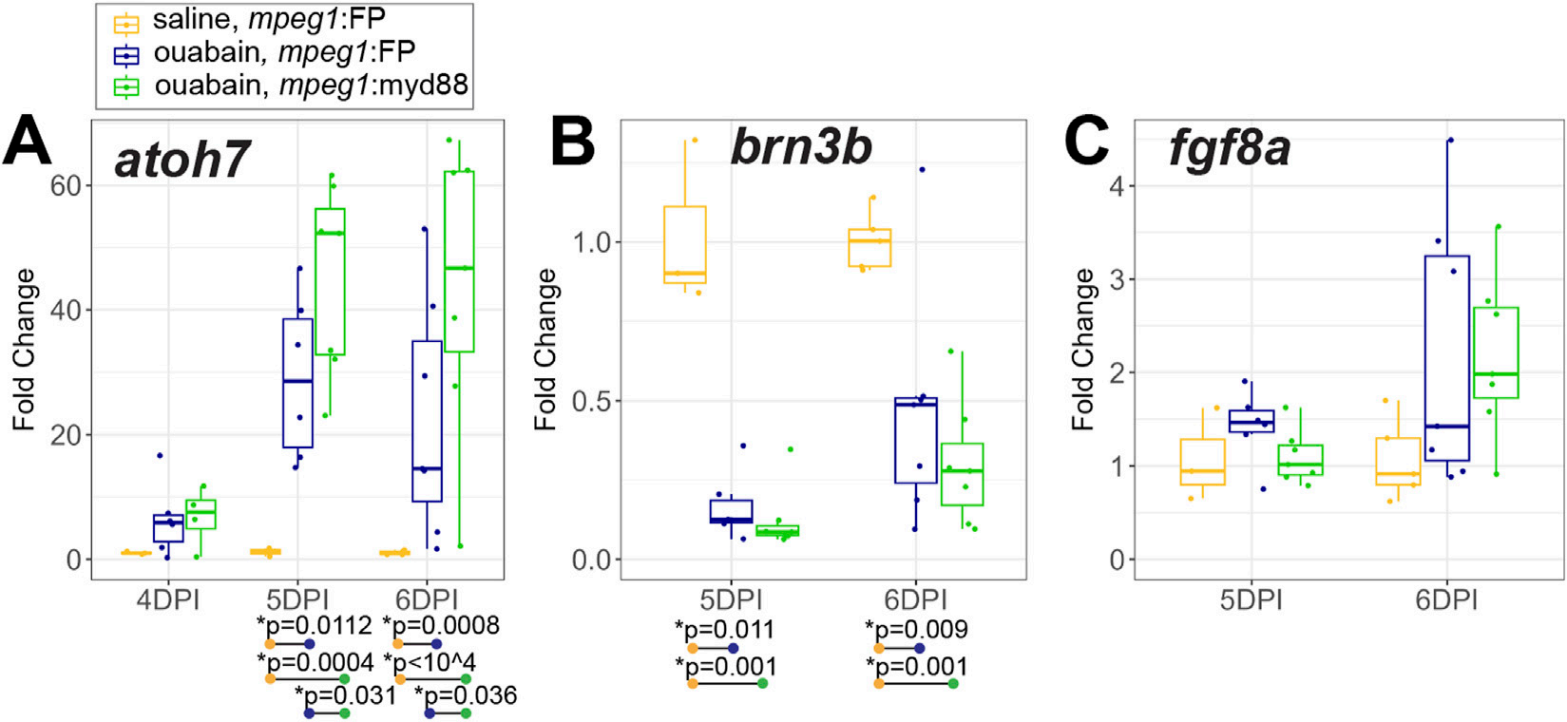
Analysis of transcripts associated with ganglion cell neurogenesis. A-C. RT-qPCR was used to examine expression of genes *atoh7* **(A)**, *brn3b* **(B)**, and *fgf8a* **(C)**, which are associated with ganglion cell neurogenesis, in *mpeg1*:FP and *mpeg1*:myd88 retinas at 4-, 5-, or 6-days post ouabain injection (4-6DPI). Fold change was determined relative to saline injected samples. Statistically significant differences between groups are shown by the *p*-values reported at the bottom of the plot (Kruskal–Wallis, followed by Conover’s posthoc). Differences in fgf8a were not statistically significant for any comparisons.

We assessed early production of regenerated inner retinal neurons at 4-6DPI by examining selected genes known to drive ganglion cell differentiation, along with markers of differentiated ganglion cells (Figure 7). RT-qPCR showed induction of the transcription factor *atoh7*, which is known to be important for ganglion cell neurogenesis (Kay et al., 2001), in both *mpeg1*:FP and *mpeg1*:myd88 retinas (Figure 7A). Expression is initially increased at 4DPI, then further and strongly increased at 5 and 6DPI consistent with the initiation of ganglion cell neurogenesis (Figure 7A). Interestingly, levels of *atoh7* were higher in *mpeg1*:myd88 retinas compared to *mpeg1*:FP at 5 and 6DPI (Figure 7A). However, when we examined levels of the genes *brn3b* and *fgf8a,* which are expressed in specific subsets of differentiated or newly generated ganglion cells, respectively (Picker and Brand, 2005; Martinez-Morales et al., 2005; Mu et al., 2004; Erkman et al., 1996; Gan et al., 1996), levels of these genes were not different between *mpeg1*:FP and *mpeg1*:myd88 samples (Figures 7B,C).

**FIGURE 8:**
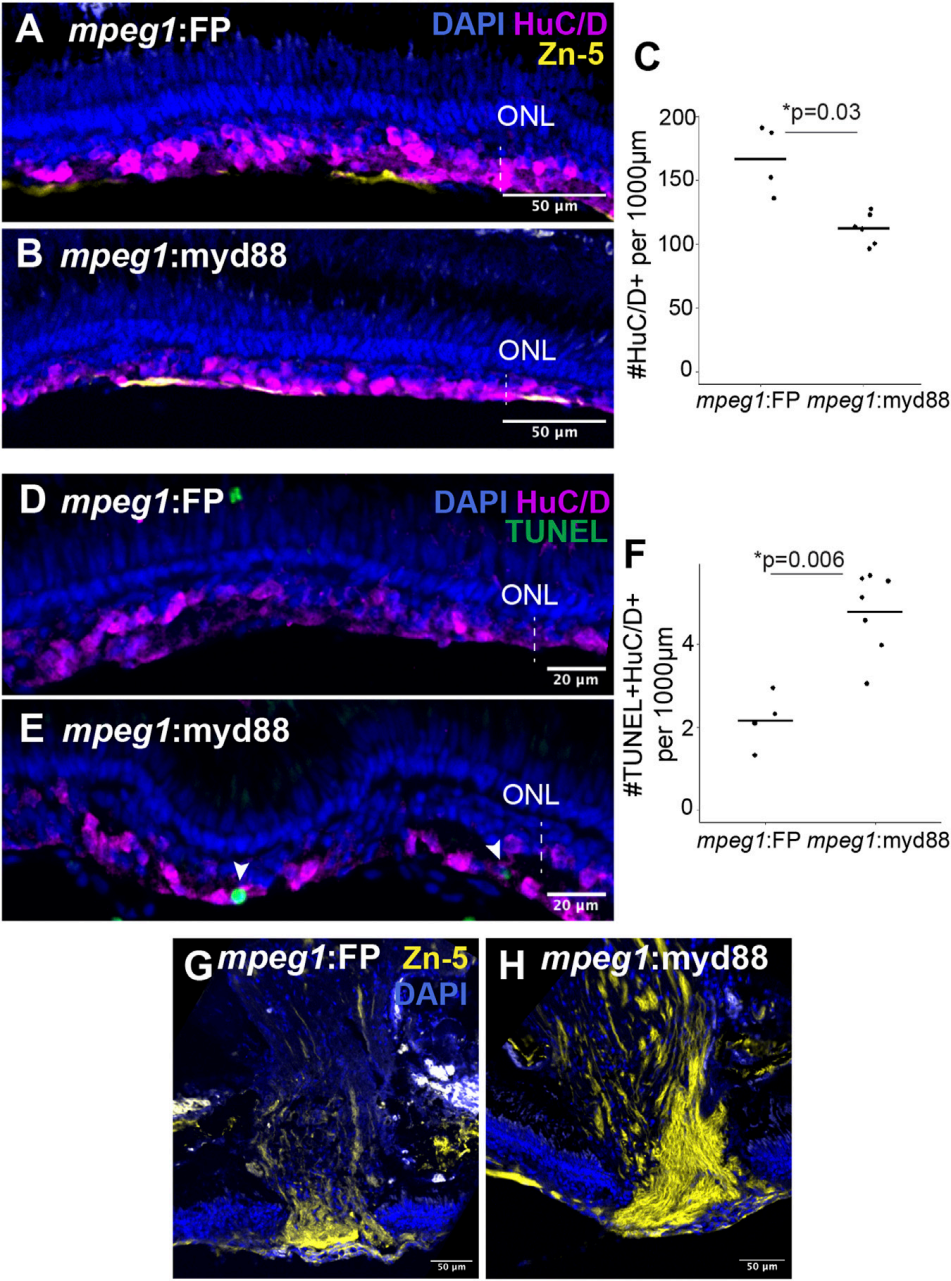
Analysis of regeneration of inner retinal neurons. **(A, B)** Retinal cryosections at 10 days post ouabain injection (10DPI) stained for HuC/D, Zn-5, and DAPI. ONL = outer nuclear layer; the vertical dotted line indicates inner retinal region damaged by ouabain. **(C)** Quantification of HuC/D+ neurons at 10DPI. Statistically significant difference between groups is indicated by the *p*-value shown at the top of the plot (Welch’s test). **(D, E)** Retinal cryosections at 10 days post ouabain injection (10DPI) stained for HuC/D, TUNEL, and DAPI. Arrowheads: TUNEL+ HuC/D+ cells. **(F)** Quantification of HuC/D+ neurons that are TUNEL+. Statistically significant differences between groups are indicated by the *p*-value shown at the top of the plot (Welch’s test). **(G, H)** Regions of regenerated optic nerve head in retinal cryosections after staining with Zn-5 antibody.

We next stained retinal cryosections for the marker HuC/D to label selected populations of regenerated inner retinal neurons, since HuC/D is expressed in differentiated ganglion cells and amacrine cells (Fimbel et al., 2007; Marusich et al., 1994). Though HuC/D+ neurons were detected at 6DPI as previously reported following ouabain lesion (Nagashima et al., 2013), their distributions were highly irregular with regions completely lacking HuC/D+ neurons, regions with sparse labeling, and regions with dense clusters making quantifications of HuC/D+ cells unreliable at this timepoint (Supplementary Figure S4). By 10DPI, HuC/D+ cells were more consistently detected in the basal regenerating inner retina of both *mpeg1*:FP and *mpeg1*:myd88 samples (Figures 8A,B). Quantification of HuC/D+ neurons at 10DPI revealed that these putatively regenerated neurons were reduced in number in *mpeg1*:myd88 retinas compared to *mpeg1*:FP (Figure 8C). To examine death of neurons at 10DPI, which could provide an explanation for these differences, we quantified the number of TUNEL+ cells in the inner retina. As described previously, cell death levels were not significantly different between groups at 2 or 6DPI (Figures 4C,F), and when examined over time both groups showed a similar trend where TUNEL+ counts were elevated upon ouabain injection then reduced over time (Supplementary Figure S5). However, at 10DPI, total numbers of TUNEL+ cells in the inner retina were increased in *mpeg1*:myd88 retinas compared to *mpeg1*:FP (Supplementary Figure S5), indicating that cell death increases upon or after the generation of new neurons in *mpeg1*:myd88 retinas. Consistent with this, regenerated retinas from *mpeg1*:myd88 fish had increased numbers of TUNEL+ HuC/D+ neurons compared to *mpeg1*:FP (Figures 8D–F). Further, in undamaged retinas from either line, TUNEL+ HuC/D+ neurons were not significantly detected, and HuC/D+ cell counts were similar (Supplementary Figure S6), again indicating that increased numbers of TUNEL+ HuC/D+ neurons in the *mpeg1*:myd88 line arise during regeneration.

Staining retinal cryosections with the antibody Zn-5 showed more intense and denser staining of Zn-5+ axon bundles in the regenerating optic nerve heads of *mpeg1*:myd88 retinas compared to *mpeg1*:FP (Figures 8G,H). Since the antigen detected by Zn-5 antibody (Alcama, also called neurolin, DM-GRASP) is expressed in active ganglion cell axon outgrowths (Karlstrom et al., 1996; Fashena and Westerfield, 1999) this suggests ganglion cell axon outgrowth is more active at 10DPI in *mpeg1*:myd88 retinas compared to *mpeg1*:FP.

Collectively, the results for HuC/D+ neuron counts (Figure 8C) and cell death counts (Figure 8F) suggest that the survival of regenerated neurons is compromised when microglia/macrophages have sustained *myd88* signaling. The increased axon outgrowth staining also seen in *mpeg1*:myd88 retinas (Figures 8G,H) could indicate delay in axon regeneration from regenerated ganglion cells, either due to direct effects of MyD88-driven signals from microglia and/or due to temporally delayed production of ganglion cells to compensate for their reduced survival.

## Discussion

In this paper, we examined the effects of forced MyD88 expression in zebrafish microglia/macrophages on retinal damage and Müller glia mediated regeneration after neurotoxin-induced retinal lesion. A transgenic line was generated to drive *myd88* expression in *mpeg1*+ cells, which in zebrafish represent microglia and macrophages (Mitchell et al., 2019; Oosterhof et al., 2017; Ellett et al., 2011; Mitchell et al., 2018). Compared to *mpeg1*:FP, we found that in retinas with forced *mpeg1*:myd88 expression, acute damage and early leukocyte responses were not changed at 2 days post retinal lesion (2DPI). However, at 4DPI we found evidence of an initial reduction in MG reactivity and in the induction of the key stem-like gene *ascl1a*, suggesting that the increased activation of the MyD88 pathway in *mpeg1*+ cells at least partially impacts the transition of Müller glia into a regenerative response. By 6DPI, *ascl1a* expression in *mpeg1*:myd88 retinas had matched or even surpassed that of *mpeg1*:FP and PCNA+ MG derived progenitors were possibly more abundant than *mpeg1*:FP. Such results suggest that the *mpeg1*:myd88 retinas were more actively producing proliferating progenitors at 6DPI than *mpeg1*:FP.

Perhaps seemingly conflicting with such a conclusion was increased *atoh7* mRNA at 6DPI in *mpeg1*:myd88 *versus mpeg1*:FP retinas. Atoh7 (also called Math5) is a key driver of ganglion cell (GC) neurogenesis (Kay et al., 2001; Brzezinski et al., 2012) and our results could first be thought to suggest that production of regenerated retinal neurons is increased in *mpeg1*:myd88 samples. However, Atoh7 is also expressed in neuronal progenitors in the retina prior to ganglion cell specification (Brzezinski et al., 2012; Brown et al., 2001; Wang et al., 2001; Yang et al., 2003) and other genes associated with ganglion cell neurogenesis and differentiation, *brn3b* and *fgf8a* (Picker and Brand, 2005; Martinez-Morales et al., 2005; Mu et al., 2004; Erkman et al., 1996), were detected at similar levels in the two groups in our study. Further, a recent study using single cell transcriptomics found that *atoh7* is expressed in a putative progenitor population that, upon retinal damage in zebrafish, temporally arises after the production of MG/progenitor “hybrids” but before the ganglion cell trajectory (Celotto et al., 2023). Therefore, increased *atoh7* levels at 6DPI could instead represent a higher abundance of these “late” progenitors rather than increased ganglion cell neurogenesis.

When we examined HuC/D+ neurons in regenerated retinas at 10DPI, we detected reduced numbers in *mpeg1*:myd88 retinas compared to *mpeg1*:FP. Further, we found in the *mpeg1*:myd88 samples that more of the HuC/D+ neurons in regenerated retinas were also positive for TUNEL, indicating that their survival was compromised. Compromised survival of regenerated neurons in the presence of prolonged inflammatory signals is consistent with the conclusions from a previous study that examined the role of a matrix metalloproteinase expressed by Müller glia upon retinal damage (Silva et al., 2020). In addition, loss of MyD88 function was found to improve photoreceptor survival in a mouse model of retinal degeneration (Syeda et al., 2015); this effect could possibly be explained through modulation of cellular chaperone activity (Carmy‐Bennun et al., 2021). The *mpeg1*:myd88 line had increased Nfkb signaling after ouabain-induced damage, but it is possible that even though induction of the MyD88 signaling pathway is ligand-dependent, forced expression of MyD88 could lead to low-level activation of this pathway in the basal state that may have some effects on regenerated cells and tissue. Low-level activation of MyD88 signaling could also potentially underlie the low-level of transgene expression in adult retinas in the undamaged state, as this could represent an attempt to reduce activation of this pathway in the absence of ligand. Nonetheless, our results indicate that microglia phenotype can impact neuronal survival in regenerated retinas, though it is not clear whether this is mediated through direct signaling or indirect mechanisms that may alter the cellular environment, progenitors, differentiating neurons, and/or other cell types.

We also found evidence of increased levels of active GC axon outgrowths in the regenerating optic nerve head of *mpeg1*:myd88 retinas compared to *mpeg1*:FP. Since the molecule recognized by the Zn-5/8 antibody is transiently expressed during active outgrowth, we considered that this increase in staining may represent axons from regenerated, but temporally-delayed, ganglion cells. When considering these results, the increased *atoh7* levels could alternatively, or in addition, represent delayed compensatory production of ganglion cells due to reduced survival of the regenerated neurons. If this is the case, then this would suggest that additional mechanisms are in place for “sensing” of neuronal replacement in the regenerating zebrafish retina. Thus, it is possible that the slight increase in *ascl1a* and proliferation of MG-derived progenitors at 6DPI also represents compensatory neurogenesis because of reduced survival of regenerated neurons.

Whatever the case, the increased MyD88 expression in microglia/macrophages resulted in trends suggesting a partial delay in reactivity/stem-like gene induction in the MG and in compromised survival of inner retinal neurons in regenerated retinas. These effects could possibly occur through intercellular signaling, where prolonged/increased activation of the MyD88 pathway in microglia/macrophages results in increased (or decreased) signaling to other cells in the damaged and regenerating retina. It is not clear if this would involve direct microglia/macrophage to Müller glia signaling or if other cell types are involved in such crosstalk (Mitra et al., 2022). Using the Nfkb::gfp reporter indicated that other retinal cell types besides leukocytes have increased Nfkb transcriptional activity in the *mpeg1*:myd88 retinas, further supporting intercellular induction of inflammatory signaling. However, we cannot be certain if this results from prolonged activation of signals that normally take place, or if other signals/pathways are triggered in other cell types. It is also not clear if similar results would be found in different retinal/CNS damage models.

Though manipulations of the MyD88 pathway in this study were intended to increase inflammatory signaling in microglia, it is worth considering that inflammatory signals can trigger anti-inflammatory responses as a mechanism for promoting a balanced outcome. Our finding of elevated *il10* expression in *mpeg1*:myd88 retinas could implicate such a counterbalance. Though IL-10 is considered an anti-inflammatory cytokine, reports in the literature indicate that this cytokine could function in unanticipated ways depending on context (Lu and Hyde, 2024; Sanchez-Molina et al., 2022; Cockey et al., 2021). Further, we did not assess cytokine expression at earlier timepoints to 4DPI, as isolation of ouabain-lesioned retinal tissue for RNA extraction prior to 4DPI is unreliable due to tissue degradation. It is therefore possible that expression patterns of inflammatory cytokines are increased earlier in *mpeg1*:myd88 retinas. In addition to cytokines, other factors such as extracellular matrix modifying enzymes are regulated by inflammatory signaling and therefore conceivable that the observed outcomes could potentially arise from changes to the extracellular matrix or microenvironment. This motivated us to briefly examine expression of selected *mmp* genes, where our data suggested a potential increase in *mmp13a* in *mpeg1*:myd88 retinas at 4DPI, though this result requires further validation. Regardless of cytokine or MMP expression patterns, proper experimental interrogation in future studies is required to directly determine cell types expressing the cytokines or enzymes, and to determine if these factors underlie the observed effects on regenerative responses.

Also of interest was the heterogeneous detection of activity of the Nfkb::gfp reporter in leukocytes at 4 days post retinal lesion. This could perhaps represent different capacity for activating Nfkb-mediated transcriptional responses in subsets of microglia/macrophages which could even be temporally dynamic. Alternatively, this could simply represent inherent differences in reporter expression due to the transgene’s chromosomal integration and/or due to the minimal promoter used to drive *gfp* in this line. Consistent with heterogenous activation of signaling pathways in subsets of microglia/macrophages, we noted that *myd88* and several MyD88/Nfkb pathway components (*irak4, rel, nfkbiaa/ab/e/z*) appeared heterogeneously expressed by populations annotated as microglia in a published single cell RNA dataset produced from adult zebrafish retina (Santhanam et al., 2023). However, Palazzo et al. (Palazzo et al., 2022) reported minimal detection of an Nfkb transcriptional reporter in microglia in the mouse retina upon retinal damage which was thought to be due to the minimal promoter used to drive the reporter. Given that heterogeneity in cell type responses is becoming more appreciated, it could be worth investigating differences in subsets of microglia and macrophages that can activate, or participate in, different inflammatory signaling cascades.

Collectively, our results are consistent with a model in which inflammatory signals from microglia/macrophages have effects on the MG-mediated regenerative response and the survival of newly generated neurons. Our results are largely consistent with reports indicating that microglia and inflammation impact MG-mediated retinal regeneration in zebrafish (Zhang et al., 2020; White et al., 2017; Silva et al., 2020; Iribarne and Hyde, 2022; Bludau et al., 2024; Conedera et al., 2019). While cytokines are likely important (Zhao et al., 2014; Lu and Hyde, 2024; Nelson et al., 2013) the specific molecular drivers and their effects are probably complex, nuanced, and depend on context (Silva et al., 2020; Iribarne and Hyde, 2022; Fogerty et al., 2022). Indeed, temporal control of inflammatory signaling is likely a key component in the production of MG-derived progenitors, but our work further indicates that this also impacts the differentiation and survival of regenerated neurons.

## Data Availability

The raw data supporting the conclusions of this article will be made available by the authors, without undue reservation.
