# Supplementary material for: Forced MyD88 signaling in microglia impacts the production and survival of regenerated retinal neurons
Source: Front Cell Dev Biol. 2024 Nov 20;12:1495586. doi: 10.3389/fcell.2024.1495586 (PMC11614808; doi:10.3389/fcell.2024.1495586)
Supplement: Supplementary file 1 [file DataSheet1.pdf]

Supp. Fig 1

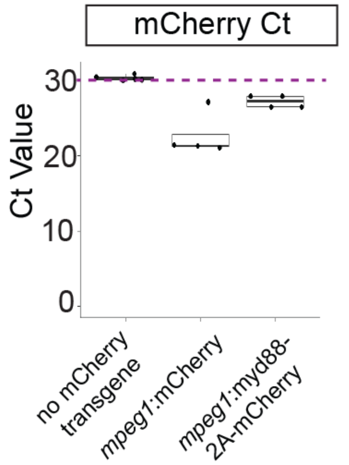

Supplemental Figure 1. Raw Ct values returned after RT-qPCR using cDNA from whole retinas from the zebrafish lines indicated on the x-axis, and primers to amplify *mCherry*. The dotted line in magenta represents the approximate limit of reliable detection. (Lower Ct values indicate more abundant transcript).

Supp. Fig 2

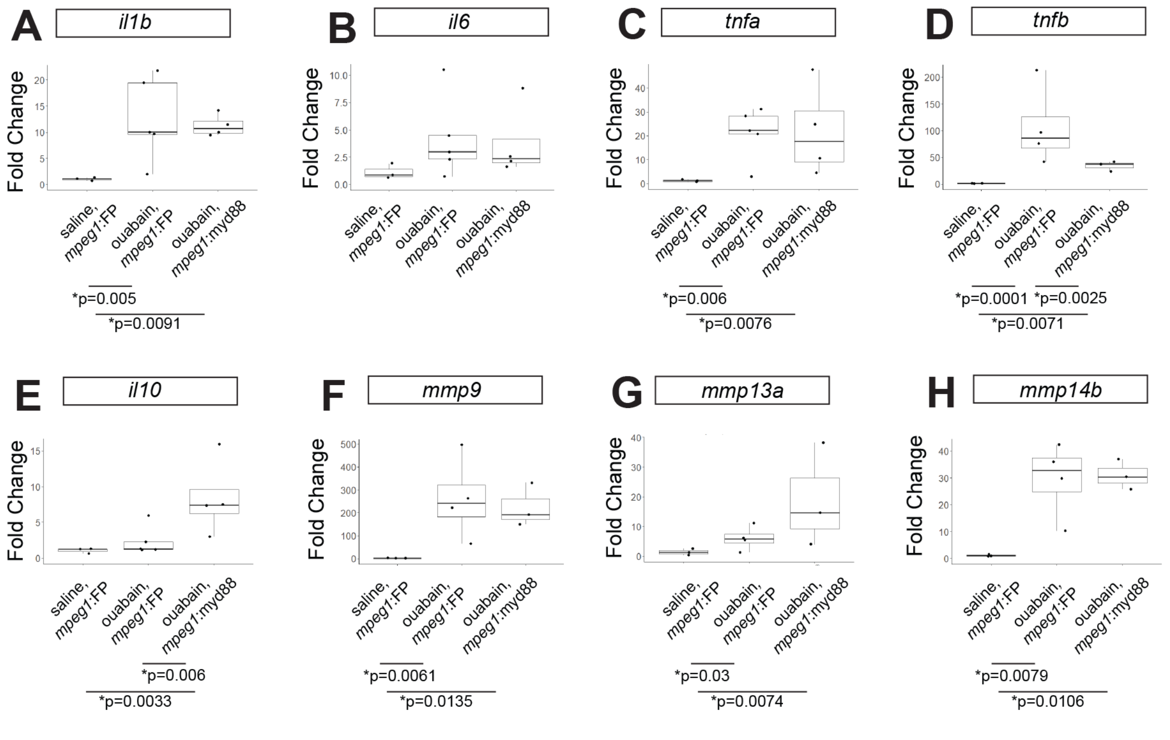

Supplemental Figure 2. RT-qPCR was used to measure expression of the indicated cytokine and *mmp* genes in whole retina RNA samples collected at 4DPI saline or ouabain injection in the lines indicated. P-values shown below the graphs indicate statistically significant differences between the indicated groups (Kruskal-Wallis, followed by Conover's posthoc).

Supp. Fig 3

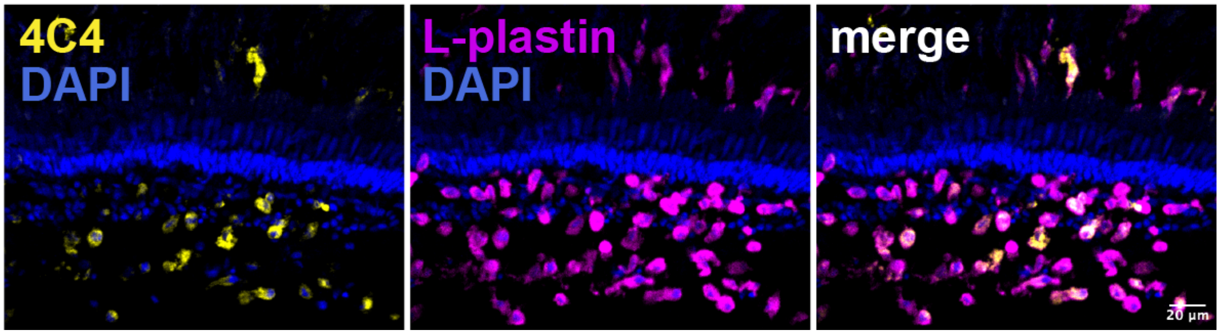

Supplemental Figure 3. Retinal cryosections from *mpeg1*:FP samples at 2 days post ouabain injection, stained with the 4C4 antibody, antibody to L-plastin, and DAPI. 4C4 staining is seen in a subset of L-plastin+ cells in acutely damaged retinas. ONL=outer nuclear layer; the vertical dotted line indicates inner retinal region damaged by ouabain.

Supp. Fig 4

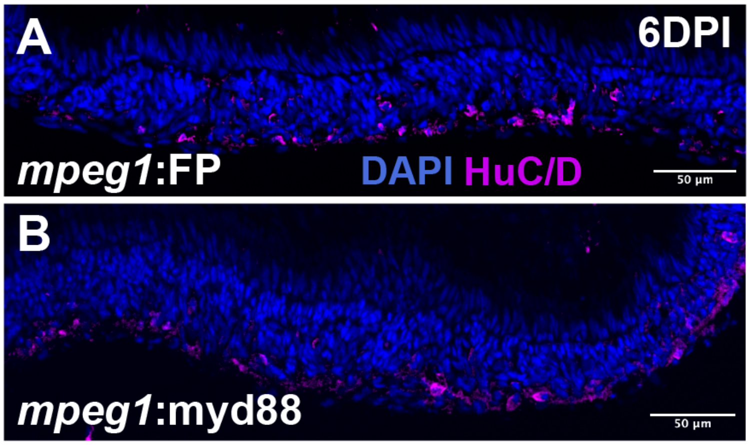

Supplemental Figure 4. A, B. Retinal cryosections at 6DPI stained for HuC/D and DAPI. ONL=outer nuclear layer; the vertical dotted line indicates inner retinal region damaged by ouabain.

Supp. Fig 5

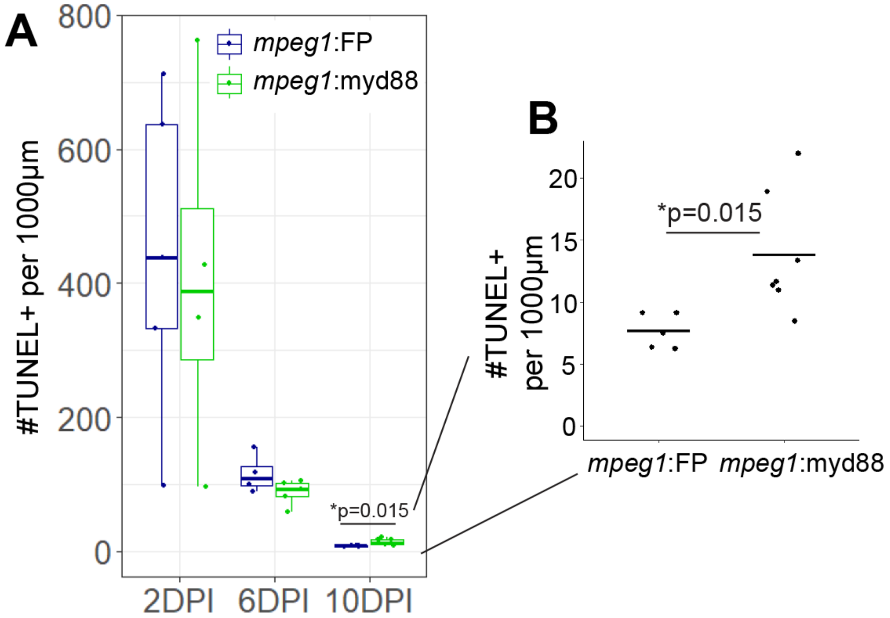

Supplemental Figure 5. A. Cell death over time, measured by TUNEL staining in retinal cryosections. B. TUNEL+ counts in retinal cryosections at 10DPI. This is the same data as shown in panel A but re-scaled to better visualize counts at this timepoint. Statistically significant differences are shown by the p-value in the plots (Welch's test).

Supp. Fig 6

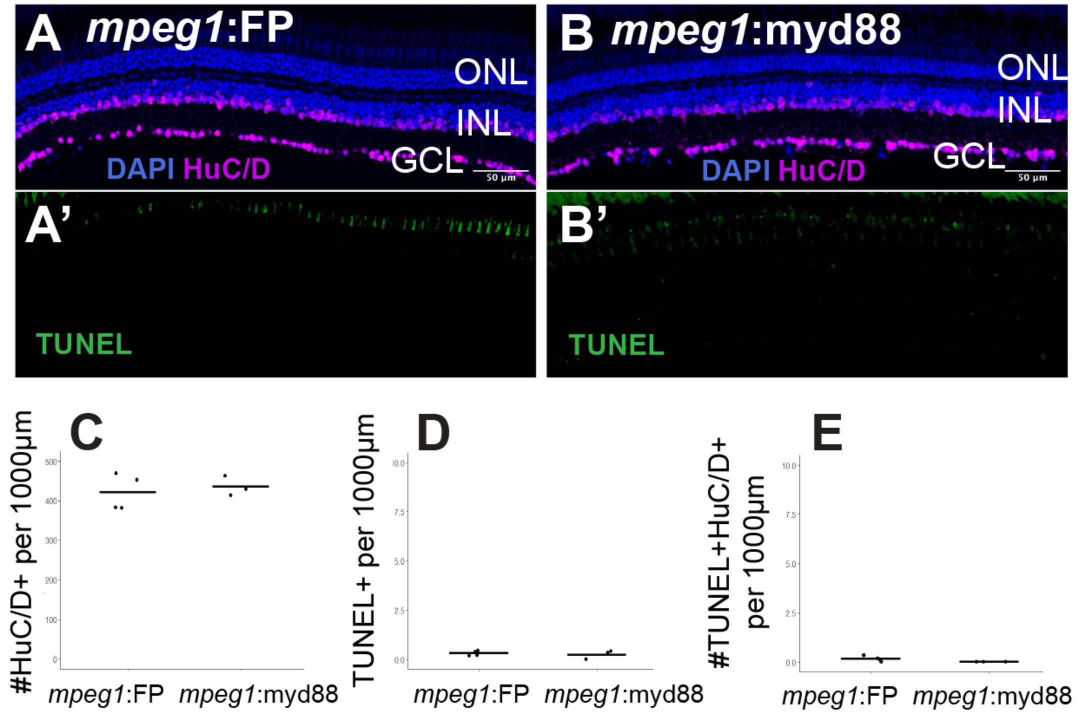

Supplemental Figure 6. Retinal cryosections from undamaged *mpeg1*:FP (A, A') or *mpeg1*:myd88 (B, B') fish eyes were stained for HuC/D, DAPI (A, B), and TUNEL (A', B'). C. Quantification of HuC/D+ neurons. D. Quantification of TUNEL+ cells. E. Quantification of TUNEL+HuC/D+ neurons. No significant TUNEL staining was detected in undamaged retinal tissue from either line. No statistically significant differences were found between *mpeg1*:FP and *mpeg1*:myd88 (Welch's test).
